# Identifying High-Yield Groups for the Detection of Low Serum Magnesium in Diabetes: A Nationally Representative Study of Korean Adults

**DOI:** 10.3390/jcm15208164

**Published:** 2026-10-20

**Authors:** Hansol Chung

**Affiliations:** Department of Emergency Medicine, Yeungnam University College of Medicine, Daegu 42415, Republic of Korea; emerhs@yu.ac.kr

**Keywords:** magnesium, hypomagnesemia, diabetes mellitus, glycemic control, kidney function, diagnostic yield, targeted testing, KNHANES

## Abstract

**Highlights:**

**What are the main findings?**

- Testing adults with diabetes who have HbA1c ≥8% or eGFR <60 mL/min/1.73 m^2^ identified low serum magnesium in about one in five tested (21.7%), twice the yield of testing all adults with diabetes (10.7%).
- This criterion required testing only 17.6% of adults with diabetes and, among guideline-based criteria with a Youden index of at least 0.20, needed the fewest tests while achieving the highest diagnostic yield.

**What are the implications of the main findings?**

- Because the criterion uses two routinely measured laboratory values, it could be applied in any clinical setting, without relying on patient-reported history, to help decide which patients with diabetes to test for serum magnesium.
- Testing these groups yields the most cases per test, enabling efficient detection of low serum magnesium in adults with diabetes.

**Abstract:**

**Background/Objectives**: Diabetes predisposes to magnesium deficiency, yet serum magnesium is not routinely measured. Using an unselected national sample, I sought a practical criterion for predicting which adults with diabetes are more likely to have low serum magnesium (<0.75 mmol/L) and examined clinical correlates. **Methods**: I analyzed 5798 adults aged ≥19 years from the 2024 Korea National Health and Nutrition Examination Survey using survey-weighted methods. Diagnostic yield was the proportion tested with low serum magnesium. Criteria combining guideline-based glycated hemoglobin (HbA1c) and estimated glomerular filtration rate (eGFR) thresholds were compared by diagnostic yield and Youden index. **Results**: Low serum magnesium occurred in 10.7% of adults with diabetes versus 2.4% without diabetes; those with diabetes accounted for 41.0% of all cases. Among criteria with a Youden index of at least 0.20, HbA1c ≥8% or eGFR <60 mL/min/1.73 m^2^ required the fewest tests and had the highest diagnostic yield (21.7%). This group comprised 17.6% of adults with diabetes but 35.7% of cases. Low serum magnesium was associated with HbA1c ≥8% (odds ratio 3.10; 95% confidence interval 1.64–5.84) and albuminuria (2.10; 1.17–3.75), largely consistently across sensitivity analyses. Exploratory analyses also identified associations with depressive symptoms and fatigue. Dietary magnesium density did not differ by magnesium status. **Conclusions**: Testing adults with diabetes who had HbA1c ≥8% or eGFR <60 mL/min/1.73 m^2^ identified low serum magnesium about twice as efficiently as testing all adults with diabetes.

## 1. Introduction

Magnesium is an essential mineral involved in more than 600 enzymatic reactions and plays key roles in glucose metabolism, insulin signaling, energy production, and neuromuscular function [1,2]. Magnesium deficiency can therefore have adverse health effects. It aggravates insulin resistance [3], and prospective evidence indicates that low serum magnesium predicts incident type 2 diabetes [4]. In patients with diabetic nephropathy, hypomagnesemia independently predicted progression to end-stage kidney disease [5]. In a national cohort of U.S. veterans with type 2 diabetes, low serum magnesium was also associated with a higher risk of major adverse cardiovascular events [6]. Fatigue and weakness are early signs of magnesium deficiency [7], and hypomagnesemia has been independently associated with depressive symptoms in older adults with diabetes [8].

Diabetes is a major cause of magnesium deficiency. Hyperglycemia and insulin resistance reduce renal magnesium reabsorption, while magnesium deficiency further impairs insulin sensitivity, creating a vicious cycle [3]. Some hospital-based studies have therefore proposed routine serum magnesium testing in patients with diabetes [9,10]. However, despite its recognized importance, serum magnesium is often not routinely assessed in clinical practice [2]. The American Diabetes Association recognizes a benefit of mineral supplementation only in the presence of an underlying deficiency [11], making the identification of deficiency important when supplementation is considered. Existing studies have largely involved single-center samples of a few hundred patients. Such studies cannot establish how common low serum magnesium is among adults with diabetes in the general population or in which patients testing would be most efficient.

Serum magnesium was measured in the 2024 Korea National Health and Nutrition Examination Survey (KNHANES) [12]. A previous analysis of these data reported an inverse association between serum magnesium and metabolic dysfunction-associated steatotic liver disease [12] but did not examine the burden or clinical correlates of low serum magnesium in adults with diabetes.

Using this nationally representative sample, I aimed to propose a practical criterion to help clinicians predict which adults with diabetes are more likely to have low serum magnesium and consider testing accordingly. Specifically, I estimated the prevalence and diagnostic yield of low serum magnesium across subgroups defined by glycemic control and kidney function and, secondarily, examined its association with poorer clinical status.

## 2. Materials and Methods

### 2.1. Data Source and Study Population

KNHANES is a nationwide cross-sectional survey conducted by the Korea Disease Control and Prevention Agency. It uses a stratified, multistage cluster probability sampling design to select a representative sample of the non-institutionalized population and comprises a health interview, a health examination, and a nutrition survey [13]. Data from 2024, the third year of the ninth survey cycle, were used.

Of the 6997 participants in the 2024 survey, 6033 were aged ≥19 years. After excluding pregnant women (*n* = 16) and participants without serum magnesium measurements (*n* = 219), 5798 adults were included (Figure 1). Of these, 959 had diabetes and 4655 did not have diabetes; glycemic status could not be determined for 184 participants.

### 2.2. Serum Magnesium

Serum magnesium was measured using a xylidyl blue colorimetric assay (Magnesium Gen.2; Roche Diagnostics, Mannheim, Germany) on a Cobas 8000 analyzer (Roche Diagnostics) and reported to one decimal place in mg/dL. Values were converted to mmol/L by multiplying by 0.4114. Low serum magnesium was classified as a recorded value ≤1.8 mg/dL (nominal threshold 0.75 mmol/L), the conventional lower limit of the reference interval [14]. The higher threshold was defined as a recorded value ≤2.0 mg/dL (nominal threshold 0.85 mmol/L) and the stricter threshold as ≤1.7 mg/dL (approximately 0.70 mmol/L). Because higher thresholds have been proposed [14,15], serum magnesium was also analyzed as a continuous variable (per 0.1 mmol/L decrease) to assess associations without relying on a specific cutoff.

### 2.3. Diabetes and Kidney Function

In accordance with the KNHANES definition, non-pregnant participants who had fasted for ≥8 h were classified as having diabetes if they met any of the following criteria: fasting plasma glucose ≥7.0 mmol/L, a physician-diagnosed history of diabetes, use of glucose-lowering medication or insulin, or glycated hemoglobin (HbA1c) ≥6.5%. Prediabetes was defined as fasting plasma glucose of 5.6–6.9 mmol/L or HbA1c of 5.7–6.4% in the absence of diabetes. HbA1c was measured by high-performance liquid chromatography (HLC-723G11; Tosoh, Tokyo, Japan), and serum and urine creatinine were measured by the Jaffe method (CREJ2; Roche Diagnostics) on a Cobas 8000 analyzer. The estimated glomerular filtration rate (eGFR) was calculated using the 2021 Chronic Kidney Disease Epidemiology Collaboration creatinine equation [16]. HbA1c ≥8% was used to define poor glycemic control because it exceeds the less stringent glycemic targets recommended for older adults and those with comorbidities [11] and corresponds to an estimated average glucose of approximately 180 mg/dL [17], near the renal threshold for glycosuria, which promotes renal magnesium loss. Reduced kidney function was defined as eGFR <60 mL/min/1.73 m^2^, the threshold for KDIGO category G3a [18]. Because kidney function was assessed using a single creatinine measurement, this category was termed reduced kidney function rather than chronic kidney disease.

### 2.4. Clinical Outcomes

Six clinical outcomes were examined in adults with diabetes: (1) poor glycemic control (HbA1c ≥8%; Section 2.3); (2) albuminuria, defined as a urine albumin-to-creatinine ratio (UACR) ≥30 mg/g in a random urine sample (urine albumin measured by immunoturbidimetry; ALBT2, Roche Diagnostics); (3) depressive symptoms, defined as a Patient Health Questionnaire-9 (PHQ-9) score ≥10 [19]; (4) fatigue, defined as a response of “more than half the days” or “nearly every day” to PHQ-9 item 4 (feeling tired or having little energy); (5) low handgrip strength, defined as a maximum measured handgrip strength (digital grip dynamometer T.K.K. 5401; Takei Scientific Instruments, Niigata, Japan) <28 kg in men or <18 kg in women [20], with measurements available only for participants aged ≥40 years; and (6) self-reported physician-diagnosed myocardial infarction or angina. Participants with urine albumin values below the lower reporting limit (3.0 mg/L) were classified as not having albuminuria. For all such participants, the maximum possible UACR based on their urine creatinine concentration was <30 mg/g, confirming this classification. Because fatigue was assessed using a PHQ-9 item, the fatigue and depressive symptom measures partly overlap.

### 2.5. Dietary Intake

Dietary magnesium and energy intakes were obtained from a single 24 h dietary recall in the nutrition survey. Dietary magnesium density was expressed as magnesium intake in mg per 1000 kcal. Dietary intakes were compared between serum magnesium groups, and the between-group difference in magnesium intake was also estimated using linear regression adjusted for age, sex, and log-transformed energy intake.

### 2.6. Covariates

Models were adjusted for age, sex, body mass index, household income quartile, use of glucose-lowering medication or insulin, and eGFR. The albuminuria model was additionally adjusted for HbA1c.

### 2.7. Statistical Analysis

All analyses, except the prediction models described below, accounted for the complex survey design, including sampling weights, strata, and clusters, and subgroup estimates were obtained using domain analysis. Examination weights were used for serum magnesium and clinical outcomes, and the combined interview, examination, and nutrition survey weight was used for dietary variables. The prevalence of low serum magnesium and its 95% confidence interval (CI) were estimated for each group. Prevalence across HbA1c categories was compared using the Rao–Scott corrected chi-square test, with a test for trend from survey-weighted logistic regression treating the categories as ordinal and pairwise comparisons from the same model; nonlinearity of the continuous association was assessed with a restricted cubic spline. Diagnostic yield was defined as the proportion of adults tested who had low serum magnesium. It quantifies case-finding efficiency only and does not address the effects of subsequent treatment. National case counts were estimated as weighted population totals. Each group’s share of the adult population and of all cases of low serum magnesium was calculated relative to the corresponding weighted total in the full sample of 5798 adults, including the 184 whose glycemic status was unavailable.

In adults with diabetes, associations between low serum magnesium and each outcome were estimated using survey-weighted logistic regression and reported as odds ratios (ORs) with 95% CIs. Multiple comparisons were addressed using the Benjamini–Hochberg procedure to control the false discovery rate across the six outcomes within each exposure definition (q < 0.05) [21]. Differences in these associations between adults with and without diabetes were assessed by including an interaction term between low serum magnesium and diabetes status in models for the full sample. This interaction test was not applicable to HbA1c ≥8%, because all participants meeting this criterion had diabetes by definition. Each outcome was analyzed among participants with available data; the proportion of adults with diabetes included ranged from 85.3% to 96.2% across outcomes and did not differ significantly by serum magnesium status (all *p* ≥ 0.13).

To assess the robustness of the main results to analytic choices, six sensitivity analyses were performed: (1) additional adjustment for dietary supplement use and antihypertensive medication; (2) exclusion of participants with UACR ≥300 mg/g to assess the influence of severe albuminuria; (3) exclusion of participants with eGFR <30 mL/min/1.73 m^2^, because reduced magnesium excretion in advanced kidney disease can increase serum magnesium [22]; (4) adjustment for age and sex only to assess the stability of estimates for outcomes with few events; (5) application of the proposed higher threshold of <0.85 mmol/L (≤2.0 mg/dL) [14,15]; and (6) application of a stricter threshold of ≤1.7 mg/dL (approximately 0.70 mmol/L), a threshold used in several studies of diabetes [23,24]. In addition, the diagnostic yield of the HbA1c ≥8% or eGFR <60 mL/min/1.73 m^2^ criterion was examined when participants were classified using the adjacent recorded values (≤1.7 and ≤1.9 mg/dL).

Among adults with diabetes, logistic regression models predicting low serum magnesium were developed and internally validated using 500 bootstrap resamples. Because these models were intended to assess individual-level discrimination rather than to produce population estimates, they were fitted without survey weights. Models were fitted among adults with diabetes who had complete data for all model variables (complete-case analysis; 894 of 959, 93.2%); the 65 excluded participants lacked data mainly on body mass index (*n* = 32) or alcohol intake (*n* = 30), and the same 894 participants were used for all three models. All candidate variables were entered simultaneously without stepwise selection, and continuous variables were modeled as linear terms. For internal validation, each model was refitted in each of 500 bootstrap samples and applied to the original sample; the mean difference in model performance between the bootstrap and original samples (optimism) was subtracted from the apparent AUC, and the calibration slope was estimated as the mean slope obtained by applying the bootstrap models’ linear predictors to the original sample. Calibration was also assessed with the Hosmer–Lemeshow test across deciles of predicted risk. In addition, testing criteria combining guideline-based thresholds for HbA1c (7.0, 7.5, 8.0, and 9.0%) and eGFR (90, 60, and 45 mL/min/1.73 m^2^) [11,18,25,26] were evaluated by survey-weighted diagnostic yield, sensitivity, specificity, and Youden index. Analyses were performed using R version 4.3.3 with the survey package version 4.2.1 [27].

## 3. Results

### 3.1. Characteristics of Adults with Diabetes

The 959 adults with diabetes had a mean age of 62.0 years, and 40.9% were women (Table 1). The mean HbA1c was 6.88%, 6.5% had an eGFR <60 mL/min/1.73 m^2^, and the mean serum magnesium concentration was 0.849 mmol/L. Compared with participants without low serum magnesium (*n* = 853), those with low serum magnesium (*n* = 106) were similar in age, sex distribution, and body mass index but had higher HbA1c (7.25% vs. 6.84%, *p* = 0.012), a higher prevalence of reduced kidney function (13.0% vs. 5.8%, *p* = 0.021), and more frequent use of glucose-lowering medication (85.6% vs. 72.3%, *p* = 0.036).

Among the 932 adults with diabetes who had dietary data, those with low serum magnesium had lower magnesium intake (302 vs. 348 mg/day, *p* = 0.036) and energy intake (1661 vs. 1918 kcal/day, *p* = 0.020) than those with serum magnesium at or above the cutoff, but magnesium intake per 1000 kcal was similar between the groups (186.5 vs. 190.9 mg, *p* = 0.66). After adjustment for age, sex, and energy intake, the between-group difference in magnesium intake was −6.6 mg/day (95% CI −40.0 to 26.8; *p* = 0.70).

### 3.2. Prevalence of Low Serum Magnesium and Diagnostic Yield

The prevalence of low serum magnesium was 2.3% in adults with normoglycemia and 2.6% in those with prediabetes, compared with 10.7% (95% CI 8.7–13.1%) in adults with diabetes (Table 2; Figure 2a). Adults with diabetes represented 14.3% of all adults but 41.0% of those with low serum magnesium. Nationally, an estimated 651,000 Korean adults with diabetes (95% CI 510,000–791,000) had low serum magnesium. Among adults with reduced kidney function but without diabetes (*n* = 94), the prevalence was 5.1% (95% CI 2.1–11.7%).

Among adults with diabetes, the prevalence was 9.2% in those with HbA1c <7.0%, 8.5% in those with HbA1c of 7.0–7.9%, and 22.5% in those with HbA1c ≥8.0% (Rao–Scott *p* < 0.001; *p* for trend = 0.007; ≥8.0% vs. <7.0%, *p* = 0.001) (Table 2). Prevalence was higher with than without reduced kidney function (21.3% vs. 9.9%; *p* = 0.025), although the estimate for reduced kidney function was imprecise (95% CI 11.3–36.7%; *n* = 76). The two criteria overlapped little (13 adults met both). Among adults with diabetes meeting either criterion (HbA1c ≥8% or eGFR <60 mL/min/1.73 m^2^; *n* = 171), the diagnostic yield was 21.7% (95% CI 15.1–30.2%); these adults comprised 17.6% of adults with diabetes but 35.7% of cases, whereas the yield was 8.3% among those meeting neither criterion (Table 2; Figure 2b).

### 3.3. Low Serum Magnesium and Clinical Outcomes in Adults with Diabetes

Four of the six outcomes were significantly associated with low serum magnesium after false discovery rate correction (Table 3; Figure 3). Compared with participants with serum magnesium at or above the cutoff, those with low serum magnesium had higher odds of HbA1c ≥8% (OR 3.10, 95% CI 1.64–5.84; q = 0.003), albuminuria (OR 2.10, 95% CI 1.17–3.75; q = 0.023), depressive symptoms (OR 3.15, 95% CI 1.36–7.29; q = 0.023), and fatigue (OR 2.19, 95% CI 1.16–4.11; q = 0.023).

Analyses treating serum magnesium as a continuous variable yielded consistent findings. For each 0.1 mmol/L decrease, ORs (95% CIs) were 2.03 (1.49–2.75) for HbA1c ≥8%, 1.44 (1.11–1.87) for albuminuria, 1.97 (1.21–3.20) for depressive symptoms, and 1.50 (1.08–2.08) for fatigue. In the categorical analysis, low serum magnesium was not significantly associated with low handgrip strength (OR 1.86, 95% CI 0.82–4.26) or myocardial infarction or angina (OR 0.62, 95% CI 0.19–2.00). There was no statistical evidence that the associations differed between adults with and without diabetes (*p* for interaction, 0.14–0.77), although the interaction tests had limited power. Given the few events, partial overlap between depressive symptoms and fatigue, and inconsistent findings across sensitivity analyses, these associations should be considered exploratory.

### 3.4. Sensitivity Analyses

The associations with HbA1c ≥8% (ORs 2.42–3.49) and albuminuria (ORs 1.61–2.82) remained significant in the first five sensitivity analyses (all q ≤ 0.032; Table S1). The associations with depressive symptoms and fatigue remained significant after additional adjustment for supplement use and antihypertensive medication (OR 3.19, q = 0.017; OR 2.08, q = 0.043, respectively) and after exclusion of the four participants with eGFR <30 mL/min/1.73 m^2^, none of whom had low serum magnesium (OR 3.13, q = 0.015; OR 2.32, q = 0.015, respectively). After exclusion of 31 participants with UACR ≥300 mg/g, the ORs were 2.48 (95% CI 0.95–6.43; q = 0.094) for depressive symptoms and 2.05 (95% CI 1.06–3.97; q = 0.065) for fatigue; in age- and sex-adjusted models, the corresponding ORs were 2.55 (95% CI 1.08–5.99; q = 0.062) and 2.00 (95% CI 1.03–3.89; q = 0.062). When the higher threshold of <0.85 mmol/L was applied, the prevalence of low serum magnesium was 42.9% (95% CI 39.2–46.7%) in adults with diabetes and 26.2% (95% CI 24.7–27.9%) in those without diabetes. All four associations remained significant at this threshold (ORs 2.42 for HbA1c ≥8%, 1.61 for albuminuria, 3.15 for depressive symptoms, and 1.84 for fatigue; all q ≤ 0.017). At the stricter threshold of ≤1.7 mg/dL (approximately 0.70 mmol/L), the prevalence of low serum magnesium was 5.0% in adults with diabetes and 0.5% in those without, and adults with diabetes accounted for 60.5% of cases; the association with HbA1c ≥8% persisted (OR 3.52, 95% CI 1.68–7.39; q = 0.003), whereas the association with albuminuria was no longer significant (OR 1.92, 95% CI 0.84–4.37; q = 0.14), reflecting the small number of participants with both albuminuria and low serum magnesium at this threshold (*n* = 15). Associations with depressive symptoms, fatigue, and low handgrip strength were also significant at this threshold but were based on very few cases (Table S1). When participants were classified using the adjacent recorded values (≤1.7 and ≤1.9 mg/dL), the HbA1c ≥8% or eGFR <60 mL/min/1.73 m^2^ criterion still increased the diagnostic yield 1.7- to 2.1-fold relative to all adults with diabetes (Table S1).

### 3.5. Prediction Models and Testing Criteria

In adults with diabetes, a model including HbA1c and eGFR had an optimism-corrected AUC of 0.62; adding age, sex, BMI, and medication use increased it to 0.64, and further adding diabetes duration, high-risk drinking, and albuminuria increased it to 0.67, with a calibration slope of 0.80, indicating modest overfitting (Table S3). Among testing criteria combining guideline-based thresholds, the Youden index was highest for HbA1c ≥7.5% or eGFR <60 mL/min/1.73 m^2^ (0.220); HbA1c ≥8% or eGFR <60 mL/min/1.73 m^2^ had a similar Youden index (0.203) with fewer adults tested (17.6% vs. 23.5%) and a higher diagnostic yield (21.7% vs. 19.6%) (Table S2).

## 4. Discussion

### 4.1. Principal Findings

In this nationally representative sample of 5798 Korean adults, low serum magnesium was concentrated in adults with diabetes, who represented 14.3% of all adults but 41.0% of those with low serum magnesium. The diagnostic yield was 10.7% among all adults with diabetes and 21.7% among those with HbA1c ≥8% or eGFR <60 mL/min/1.73 m^2^. In adults with diabetes, low serum magnesium was associated with poor glycemic control and albuminuria, largely consistently across sensitivity analyses, and, in exploratory analyses, with depressive symptoms and fatigue. Magnesium intake per unit of energy did not differ by serum magnesium status.

Thus, in the general population, low serum magnesium is less common among adults with diabetes than clinic-based estimates suggest but is concentrated in subgroups that can be identified from routinely measured laboratory values.

### 4.2. Comparison with Previous Studies

Studies of clinically selected patients have reported higher estimates: 20–40% in hospital-based studies, with a pooled prevalence of 32% in a meta-analysis of 19 studies [28], and 17.8% in a U.S. veteran cohort limited to patients whose serum magnesium had been tested [6]. Estimates from unselected samples are substantially lower: 4.8% (≤0.65 mmol/L) among 680 Chinese community-dwelling adults with diabetes [29], 9.6% (<0.70 mmol/L) in a Dutch primary care cohort [23], and 10.7% in the present study (5.0% at ≤0.70 mmol/L). National surveys that measured serum magnesium in Taiwan [30], China [31], Canada [32], and the United States [33] described magnesium status or its association with diabetes, but none examined which adults with diabetes should be tested. This gap is clinically relevant because serum magnesium is not included in standard metabolic panels and is not routinely measured in clinical practice [2,33]. In Korea, population-based data were limited because serum magnesium was not measured in KNHANES until 2024 [34], while Japanese population studies have focused on dietary rather than serum magnesium [35]. East Asian clinical studies have linked low serum magnesium to albuminuria [36,37] and diabetic kidney disease progression [5,38]. To my knowledge, no previous study has quantified the distribution of low serum magnesium among adults with diabetes in an unselected population or identified subgroups with the highest diagnostic yield.

The association between low serum magnesium and HbA1c ≥8% (OR 3.10) was consistent in direction and magnitude with that reported in a primary care study (adjusted OR 2.4) [10]. The association with albuminuria agrees with the inverse association between serum magnesium and albuminuria reported in patients with diabetes [36] and with the finding that hypomagnesemia predicted end-stage kidney disease in diabetic nephropathy [5]. The association with depressive symptoms is also consistent with findings in older adults with diabetes [8]. A previous analysis of the same 2024 data reported an inverse association between serum magnesium and hyperglycemia [12]; the present study extends this finding through an analysis restricted to adults with diabetes and adjusted for kidney function. Prevalence was higher in adults with both diabetes and reduced kidney function than in those with reduced kidney function alone. This pattern is consistent with evidence that serum magnesium usually remains normal in moderate kidney disease [22], whereas diabetes contributes to renal magnesium loss. Reduced kidney function in diabetes may therefore be a marker of longer disease duration, greater disease severity, albuminuria, or medication use, rather than a direct cause of low serum magnesium.

The prevalence of low serum magnesium depends strongly on the threshold applied. I selected 0.75 mmol/L as the primary threshold because it is the conventional lower reference limit widely used in clinical practice [14,33]. A lower threshold of 0.70 mmol/L has been used in several studies of diabetes [23,24], whereas 0.85 mmol/L has been proposed to identify chronic latent magnesium deficiency [14,15]. Because serum magnesium concentrations in adults with diabetes cluster near these thresholds, raising the threshold from 0.75 to 0.85 mmol/L increased the prevalence approximately fourfold, from 10.7% to 42.9%. At both 0.70 and 0.75 mmol/L, the proposed criterion approximately doubled the diagnostic yield relative to testing all adults with diabetes; at 0.85 mmol/L, however, the increase was modest (1.3-fold) because nearly half of adults with diabetes were classified as having low serum magnesium. Thus, targeted testing is most informative for identifying serum magnesium concentrations below the conventional reference limit, whereas concentrations between 0.75 and 0.85 mmol/L are better interpreted as indicating a risk of chronic latent magnesium deficiency than overt deficiency.

### 4.3. Potential Mechanisms

In diabetes, low serum magnesium is thought to result primarily from renal magnesium loss. Osmotic diuresis due to hyperglycemia and reduced activity of the distal tubular magnesium channel TRPM6 in insulin resistance decrease magnesium reabsorption [3]. Conversely, intracellular magnesium deficiency impairs insulin receptor signaling and pancreatic β-cell function, aggravating insulin resistance [3,39]. The progressive increase in low serum magnesium with higher HbA1c is consistent with renal magnesium loss accompanying glycosuria [3], suggesting that this association may partly reflect the effects of hyperglycemia on serum magnesium.

Participants with low serum magnesium had lower total energy intake, but their magnesium intake per unit of energy was similar to that of participants with serum magnesium at or above the cutoff. This pattern suggests that processes such as renal magnesium loss may contribute to low serum magnesium beyond differences in dietary magnesium density. However, urinary magnesium was not measured, so the relative contributions of these pathways could not be determined. Dietary intake alone may therefore be insufficient to identify individuals with low serum magnesium.

The association with albuminuria may reflect magnesium loss due to glomerular and tubular injury, as well as endothelial dysfunction associated with magnesium depletion [1,5]. The associations with depressive symptoms and fatigue are biologically plausible. Magnesium is required for the biological activity of ATP, inhibits the N-methyl-D-aspartate receptor involved in regulating neuronal excitability, and contributes to neuromuscular function [1]. Deficiency may therefore contribute to fatigue and depressive symptoms through impaired energy metabolism and increased neuronal excitability. However, only 35 participants with depressive symptoms were included in the adjusted model. Fatigue was assessed using a PHQ-9 item and is therefore not independent of the depressive symptom measure. Both associations were attenuated in some sensitivity analyses, should be regarded as exploratory, and require confirmation in larger samples.

### 4.4. Implications for Practice

These findings are intended primarily for clinicians caring for adults with diabetes and for those developing testing recommendations. Testing adults with diabetes who have HbA1c ≥8% or eGFR <60 mL/min/1.73 m^2^ identifies low serum magnesium about twice as efficiently as testing all adults with diabetes, which can help clinicians decide which patients to test, given that serum magnesium is not measured routinely. A recent review concluded that serum magnesium measurement may be considered in selected patients, such as those with diabetic kidney disease [40].

Models based on routine clinical information showed limited discrimination for low serum magnesium (optimism-corrected AUC 0.62–0.67) and modest overfitting (calibration slope 0.80); these models are therefore not intended as clinical prediction tools. Diabetes duration, medication history, and alcohol intake could also inform the selection of patients for testing, but they depend on patient recall or medical records, which are often incomplete. Such history is particularly difficult to establish when patients are seen at a new institution, and patients with chronic conditions commonly receive care from multiple providers [41,42]. Albuminuria also contributed to prediction, but it is measured in only about half of adults with diabetes, limiting its practical use [43,44,45]. In contrast, HbA1c and serum creatinine are objective laboratory values measured routinely in nearly all adults with diabetes [43,46]. The criterion of HbA1c ≥8% or eGFR <60 mL/min/1.73 m^2^ therefore relies only on values available in any clinical setting; the criterion of HbA1c ≥7.5% or eGFR <60 mL/min/1.73 m^2^ is an alternative when higher sensitivity is preferred (Table S2).

The American Diabetes Association recognizes a benefit of mineral supplementation only when an underlying deficiency is present [11]. Evidence from clinical trials is mixed. In patients with type 2 diabetes and serum magnesium ≤0.74 mmol/L, supplementation significantly reduced HbA1c [47]. By contrast, a recent trial that did not select participants by magnesium status found no significant overall effect [48]. A small 6-week crossover trial in 14 insulin-treated patients with low serum magnesium also found no improvement in insulin sensitivity [49]. A meta-analysis reported heterogeneous effects [50]. For depressive symptoms, supplementation showed a benefit in older adults with type 2 diabetes and hypomagnesemia [51]. In the U.S. veteran cohort, prescribed magnesium replacement was associated with a lower cardiovascular risk among patients with hypomagnesemia but not among those with normal serum magnesium [6]. Thus, benefits have been observed mainly in patients with documented hypomagnesemia, and testing adults with diabetes who meet the proposed criterion is an efficient way to identify such patients. Whether this targeted testing strategy reduces long-term complications and is cost-effective warrants further evaluation.

### 4.5. Strengths and Limitations

This study used nationally representative serum magnesium data from KNHANES, accounted for the complex survey design, and applied false discovery rate correction and multiple sensitivity analyses.

This study has several limitations. First, the cross-sectional design precludes causal inference. Second, serum magnesium represents less than 1% of total body magnesium and may remain normal despite depletion [1,14]; serum magnesium status therefore does not necessarily reflect total body magnesium status. Serum albumin, which affects total serum magnesium concentrations, was not measured. Excluding participants with severe albuminuria did not materially alter the associations with glycemic control and albuminuria, but this analysis could not account for variation in serum albumin. Third, serum magnesium was available only as values rounded to 0.1 mg/dL. Participants with values near the cut-off may therefore have been misclassified, and the discrete scale may have attenuated associations in the continuous analyses. In addition, serum magnesium and creatinine were each measured once. Fourth, KNHANES lacks data on individual medications, including diuretics and proton pump inhibitors. Both can lower serum magnesium [52,53,54] and may be prescribed more frequently to patients with diabetes, reduced kidney function, or poor glycemic control, potentially leading to overestimation of the observed associations. Adjustment for antihypertensive use as a proxy for diuretic exposure did not change the findings but cannot replace drug-specific data. Several glucose-lowering agents, including metformin, have also been associated with lower serum magnesium [23], and sodium–glucose cotransporter 2 inhibitor use has been reported less frequently among patients with hypomagnesemia [9]. Fifth, dietary intake was based on a single 24 h recall, which captures usual intake imperfectly because of day-to-day variation; while adequate for comparing group means, it may attenuate individual-level associations, so the absence of a difference in magnesium intake per unit of energy should be interpreted cautiously. Sixth, the performance of the testing criterion should be confirmed in external populations before it is implemented in widespread clinical practice.

## 5. Conclusions

In this nationally representative sample, low serum magnesium affected about one in nine adults with diabetes. Among those with HbA1c ≥8% or eGFR <60 mL/min/1.73 m^2^, it affected about one in five, and the diagnostic yield was roughly twice that of adults with diabetes overall. Low serum magnesium was associated with poor glycemic control and albuminuria and, in exploratory analyses, with depressive symptoms and fatigue. These findings identify adults with diabetes for whom serum magnesium testing has the highest diagnostic yield. The criterion should be regarded as a preliminary proposal for identifying patient groups in whom testing may be considered, rather than as an established screening method.

## Figures and Tables

**Figure 1:**
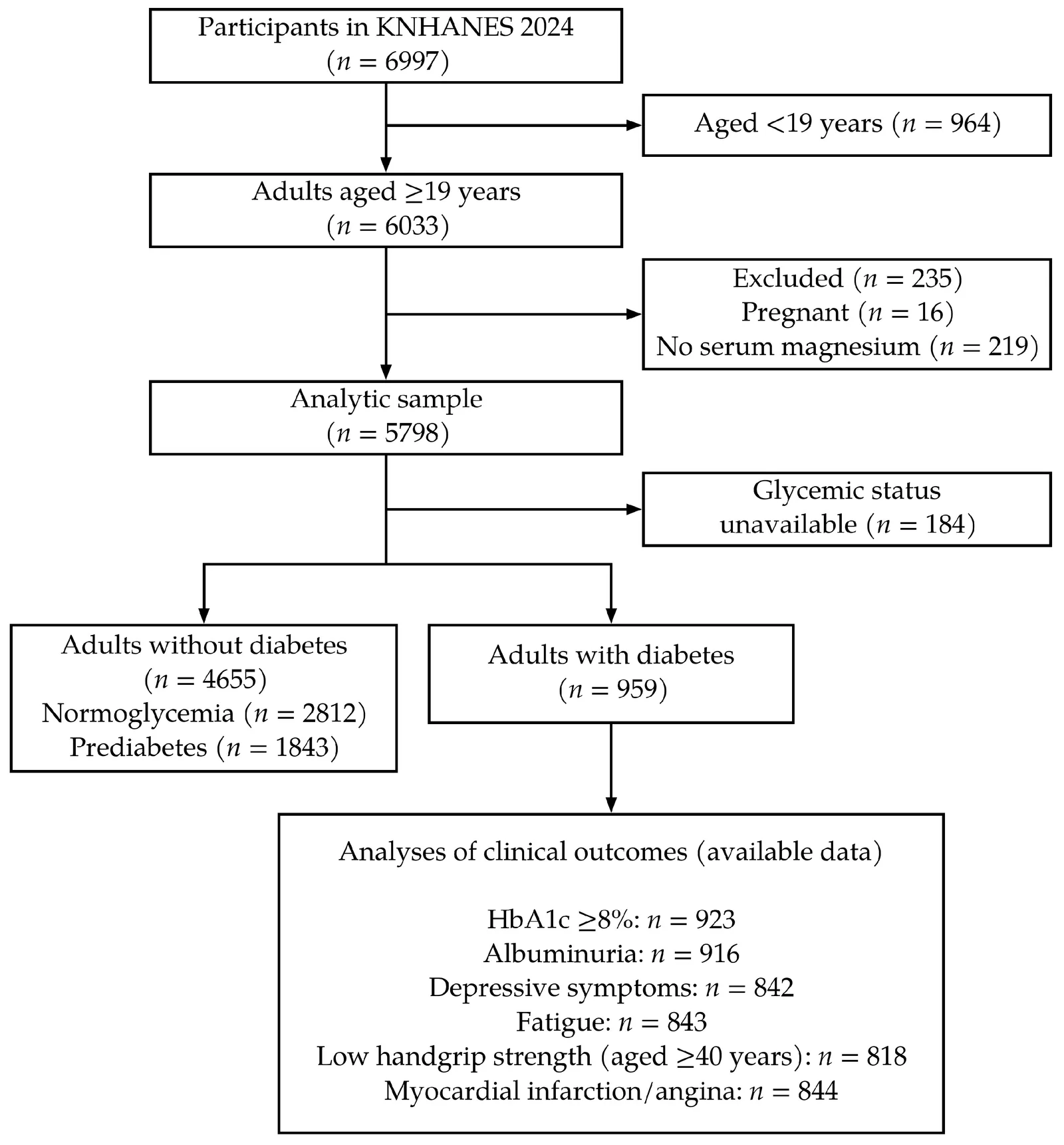
Flow diagram of participant selection. KNHANES, Korea National Health and Nutrition Examination Survey.

**Figure 2:**
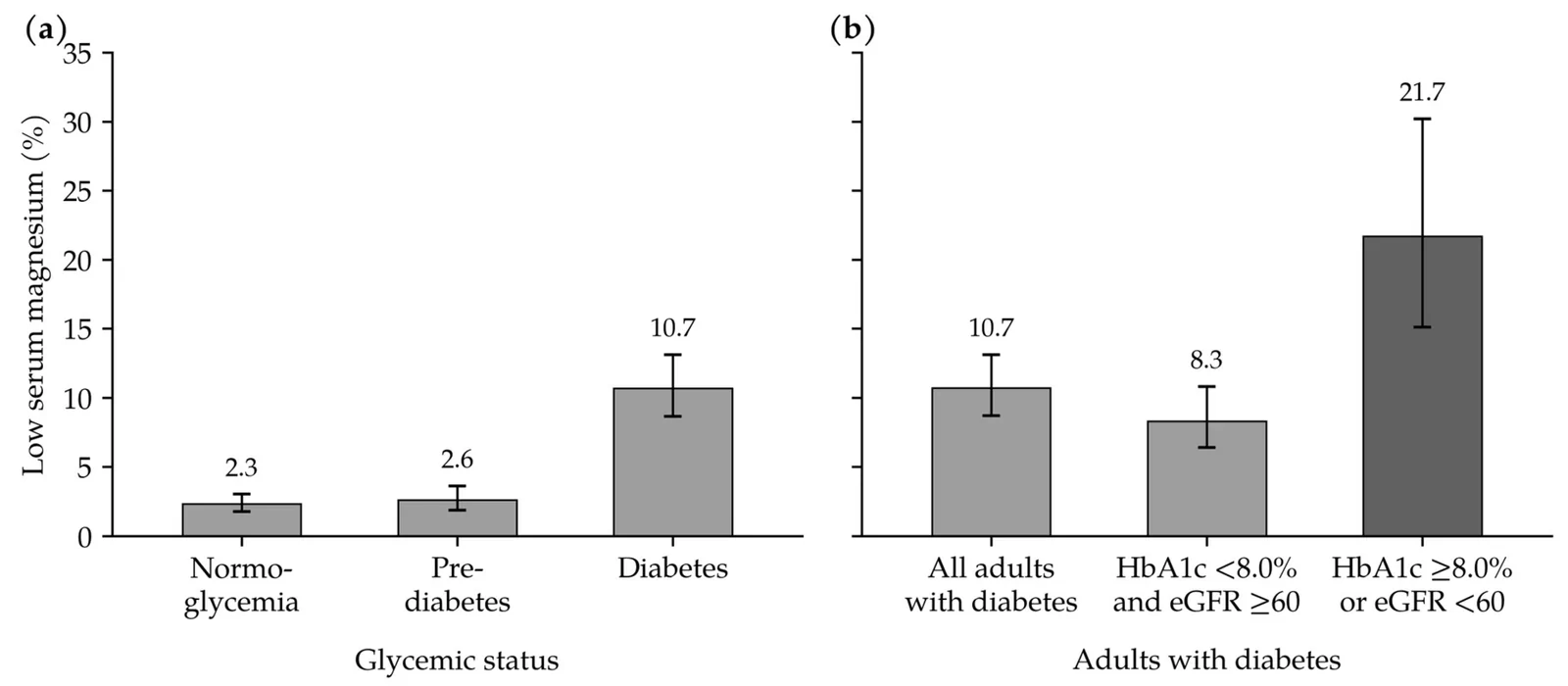
Prevalence of low serum magnesium (<0.75 mmol/L) (**a**) by glycemic status and (**b**) among adults with diabetes by the proposed testing criterion (HbA1c ≥8.0% or eGFR <60 mL/min/1.73 m^2^). Error bars indicate 95% confidence intervals from survey-weighted estimates.

**Figure 3:**
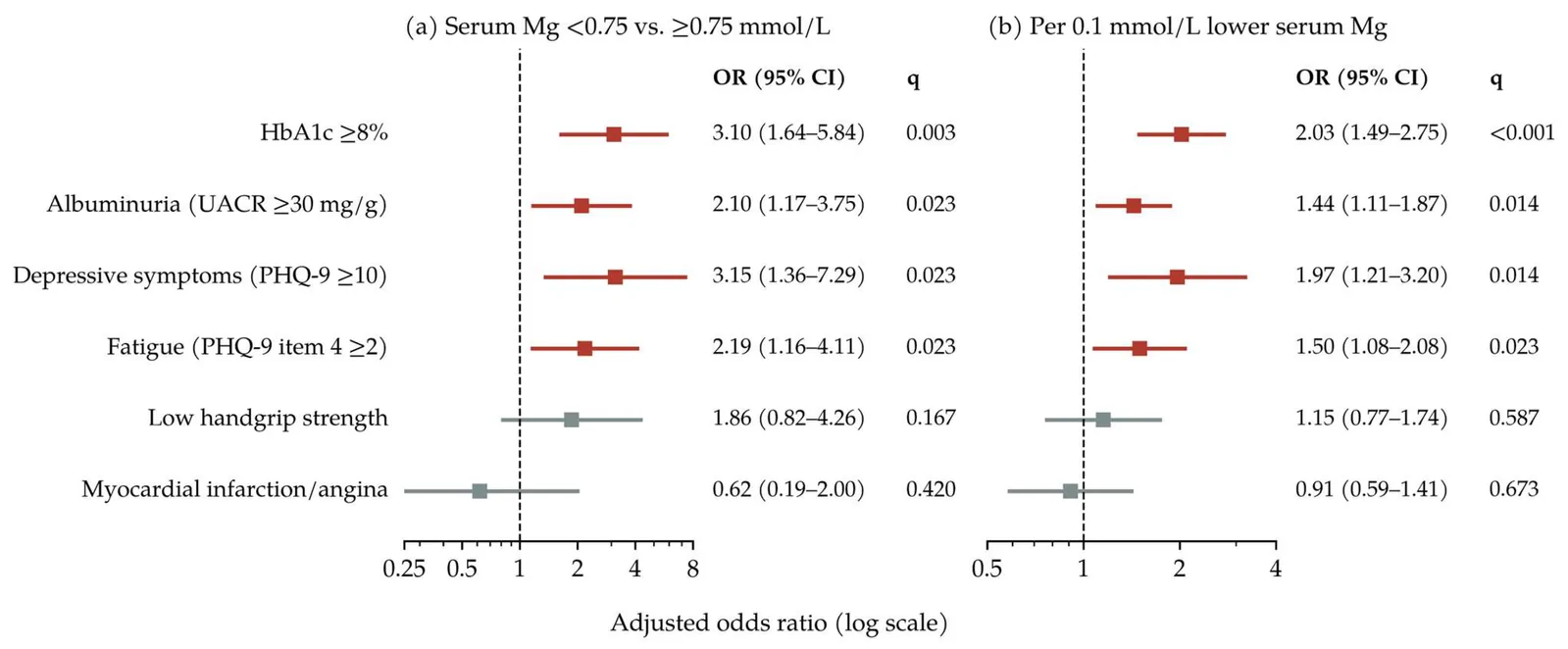
Adjusted odds ratios for clinical outcomes in adults with diabetes: (**a**) serum magnesium <0.75 vs. ≥0.75 mmol/L; (**b**) per 0.1 mmol/L lower serum magnesium. Red markers indicate q < 0.05 after false discovery rate correction; gray markers indicate q ≥ 0.05. Models are adjusted as described in Table 3. UACR, urine albumin-to-creatinine ratio; PHQ-9, Patient Health Questionnaire-9.

**Table 1:** Characteristics of adults with diabetes by serum magnesium status.

Characteristic	Total	Serum Magnesium ≥0.75 mmol/L	Serum Magnesium <0.75 mmol/L	*p*
Unweighted *n*	959	853	106	
Age, years	62.0 (0.6)	62.1 (0.6)	61.6 (1.5)	0.74
Women, %	40.9 (1.7)	41.1 (1.8)	39.4 (4.5)	0.73
BMI, kg/m^2^	25.8 (0.2)	25.8 (0.2)	25.8 (0.5)	0.95
Lowest income quartile, %	24.9 (1.8)	24.2 (1.8)	31.1 (5.1)	0.15
HbA1c, %	6.88 (0.05)	6.84 (0.05)	7.25 (0.16)	0.012
eGFR, mL/min/1.73 m^2^	89.4 (0.7)	89.9 (0.8)	85.5 (2.7)	0.13
eGFR <60 mL/min/1.73 m^2^, %	6.5 (0.8)	5.8 (0.8)	13.0 (3.8)	0.021
Glucose-lowering medication, %	73.7 (1.9)	72.3 (2.0)	85.6 (4.7)	0.036
Antihypertensive medication, %	53.4 (2.1)	52.8 (2.2)	58.5 (5.2)	0.31
Dietary supplement use, %	66.4 (1.8)	66.4 (1.9)	66.5 (5.2)	0.99
Dietary magnesium intake, mg/day ^a^	344 (7)	348 (8)	302 (20)	0.036
Energy intake, kcal/day ^a^	1891 (38)	1918 (40)	1661 (106)	0.020
Magnesium intake per 1000 kcal, mg ^a^	190.5 (2.7)	190.9 (2.8)	186.5 (9.8)	0.66
Serum magnesium, mmol/L	0.849 (0.003)	0.866 (0.002)	0.703 (0.006)	<0.001
HbA1c ≥8%, %	12.4 (1.4)	10.8 (1.3)	26.2 (5.5)	<0.001
Albuminuria (UACR ≥30 mg/g), %	19.4 (1.4)	17.4 (1.3)	36.8 (5.9)	<0.001
Depressive symptoms (PHQ-9 ≥10), %	4.8 (0.9)	4.2 (0.9)	10.1 (3.3)	0.024
Fatigue (PHQ-9 item 4 ≥2), %	10.5 (1.2)	9.7 (1.3)	17.5 (4.3)	0.038
Low handgrip strength, %	10.0 (1.1)	9.4 (1.2)	15.5 (4.1)	0.08
Myocardial infarction/angina, %	6.5 (0.9)	6.6 (0.9)	5.1 (2.3)	0.58

Values are weighted means (SEs) or weighted percentages (SEs); *n* is unweighted. *p* values were obtained from design-based tests (survey-weighted regression for continuous variables and the Rao–Scott chi-square test for categorical variables). ^a^ Based on a single 24 h dietary recall in participants with valid dietary data (*n* = 932), using the combined survey weight. BMI, body mass index; eGFR, estimated glomerular filtration rate; UACR, urine albumin-to-creatinine ratio; PHQ-9, Patient Health Questionnaire-9.

**Table 2:** Prevalence of low serum magnesium, diagnostic yield, and estimated national burden by glycemic and kidney function status.

Group	*n*	% of Adults	Prevalence, % (95% CI)	% of All Cases	Estimated Number, ×1000 (95% CI)
Adults without diabetes	4655	82.5	2.4 (2.0–3.0)	53.5	850 (670–1030)
Normoglycemia	2812	52.9	2.3 (1.8–3.0)	32.8	521 (376–667)
Prediabetes	1843	29.6	2.6 (1.9–3.6)	20.7	329 (222–436)
Reduced kidney function without diabetes	94	1.2	5.1 (2.1–11.7)	1.6	25 (3–47)
Adults with diabetes	959	14.3	10.7 (8.7–13.1)	41.0	651 (510–791)
By HbA1c					
<7.0%	655	9.6	9.2 (7.0–12.0)	23.6	374 (270–479)
7.0–7.9%	196	2.9	8.5 (5.2–13.5)	6.7	106 (52–160)
≥8.0%	108	1.8	22.5 (14.5–33.2)	10.7	170 (90–250)
By eGFR, mL/min/1.73 m^2^					
≥60	883	13.4	9.9 (7.9–12.4)	35.6	566 (432–700)
<60	76	0.9	21.3 (11.3–36.7)	5.3	85 (34–136)
By HbA1c and eGFR combined					
HbA1c <8.0% and eGFR ≥60	788	11.8	8.3 (6.4–10.8)	26.3	419 (304–533)
HbA1c ≥8.0% only	95	1.6	21.9 (13.9–32.9)	9.3	147 (76–219)
eGFR <60 only	63	0.7	19.7 (10.6–33.7)	3.9	62 (25–99)
HbA1c ≥8.0% and eGFR <60	13	0.2	27.1 (NE)	1.5	23 (0–58)
HbA1c ≥8.0% or eGFR <60 (proposed criterion)	171	2.5	21.7 (15.1–30.2)	14.6	232 (146–318)

Low serum magnesium: <0.75 mmol/L (recorded value ≤1.8 mg/dL). Prevalence corresponds to the diagnostic yield of testing. The first four rows of the combined classification are mutually exclusive; the proposed criterion row combines the three groups meeting at least one criterion. Percentages of adults and cases are relative to all 5798 adults, including 184 with undetermined glycemic status. Estimates are survey-weighted; *n* is unweighted. CI, confidence interval; eGFR, estimated glomerular filtration rate; NE, not estimable.

**Table 3:** Associations of low serum magnesium with clinical outcomes in adults with diabetes.

Outcome	Events/*n*	Serum Magnesium <0.75 vs. ≥0.75 mmol/L, OR (95% CI)	q	Per 0.1 mmol/L Lower Serum Magnesium, OR (95% CI)	q	*p* for Interaction
HbA1c ≥8%	101/923	3.10 (1.64–5.84)	0.003	2.03 (1.49–2.75)	<0.001	NA
Albuminuria (UACR ≥30 mg/g)	172/916	2.10 (1.17–3.75)	0.023	1.44 (1.11–1.87)	0.014	0.77
Depressive symptoms (PHQ-9 ≥10)	35/842	3.15 (1.36–7.29)	0.023	1.97 (1.21–3.20)	0.014	0.14
Fatigue (PHQ-9 item 4 ≥2)	83/843	2.19 (1.16–4.11)	0.023	1.50 (1.08–2.08)	0.023	0.21
Low handgrip strength	95/818	1.86 (0.82–4.26)	0.167	1.15 (0.77–1.74)	0.587	0.60
Myocardial infarction/angina	58/844	0.62 (0.19–2.00)	0.420	0.91 (0.59–1.41)	0.673	0.74

Estimates were obtained using survey-weighted logistic regression adjusted for age, sex, body mass index, household income quartile, glucose-lowering medication, and eGFR; the albuminuria model was additionally adjusted for HbA1c. q values are Benjamini–Hochberg false discovery rate-adjusted *p* values across the six outcomes within each exposure definition. *p* for interaction tests whether the association between serum magnesium <0.75 mmol/L and each outcome differed between adults with and without diabetes in the full sample. NA, not applicable, because all participants with HbA1c ≥8% had diabetes by definition. OR, odds ratio; CI, confidence interval; UACR, urine albumin-to-creatinine ratio; PHQ-9, Patient Health Questionnaire-9.

## Data Availability

The data analyzed in this study are publicly available from the KNHANES website (https://knhanes.kdca.go.kr).

## Supplementary Materials

The following supporting information can be downloaded at: https://www.mdpi.com/article/10.3390/jcm15208164/s1, Table S1: (a) Sensitivity analyses of associations between low serum magnesium and clinical outcomes in adults with diabetes; (b) diagnostic performance of the HbA1c ≥8% or eGFR <60 mL/min/1.73 m^2^ criterion when low serum magnesium is defined by adjacent recorded cut-offs; Table S2: Diagnostic performance of guideline-based testing criteria combining HbA1c and eGFR thresholds in adults with diabetes; Table S3: (a) Prediction models for low serum magnesium (<0.75 mmol/L) in adults with diabetes (*n* = 894; 98 with low serum magnesium); (b) Adjusted odds ratios in model M3; (c) Operating characteristics of model M3 compared with the proposed criterion.

## Abbreviations

The following abbreviations are used in this manuscript:

ATP	adenosine triphosphate
AUC	area under the receiver operating characteristic curve
BMI	body mass index
CI	confidence interval
eGFR	estimated glomerular filtration rate
HbA1c	glycated hemoglobin
KDIGO	Kidney Disease: Improving Global Outcomes
KNHANES	Korea National Health and Nutrition Examination Survey
OR	odds ratio
PHQ-9	Patient Health Questionnaire-9
SE	standard error
TRPM6	transient receptor potential melastatin 6
UACR	urine albumin-to-creatinine ratio
