# Supplementary material for: Identifying High-Yield Groups for the Detection of Low Serum Magnesium in Diabetes: A Nationally Representative Study of Korean Adults
Source: J Clin Med. 2026 Oct 20;15(20):8164. doi: 10.3390/jcm15208164 (PMC13706576; doi:10.3390/jcm15208164)
Supplement: Supplementary file 1 [file jcm-15-08164-s001.zip › jcm-4645943-supplementary.pdf]

Supplementary Materials

Identifying High-Yield Groups for the Detection of Low Serum Magnesium in Diabetes: A Nationally Representative Study of Korean Adults

Table S1. (a) Sensitivity analyses of associations between low serum magnesium and clinical outcomes in adults with diabetes; (b) diagnostic performance of the HbA1c ≥8% or eGFR <60 mL/min/1.73 m² criterion when low serum magnesium is defined by adjacent recorded cut-offs.

Outcome	Additional adjustment [a]	Excluding UACR ≥300 mg/g [b]	Excluding eGFR <30 mL/min/1.73 m² [c]	Age- and sex-adjusted [d]	Threshold <0.85 mmol/L [e]	Threshold ≤1.7 mg/dL [f]
HbA1c ≥8%	3.41 (1.77–6.59); q = 0.002	3.49 (1.79–6.77); q = 0.002	3.24 (1.70–6.19); q = 0.003	2.97 (1.65–5.37); q = 0.001	2.42 (1.43–4.10); q = 0.007	3.52 (1.68–7.39); q = 0.003
Albuminuria (UACR ≥30 mg/g)	2.29 (1.24–4.23); q = 0.017	2.26 (1.21–4.20); q = 0.032	2.15 (1.20–3.86); q = 0.015	2.82 (1.60–4.95); q = 0.001	1.61 (1.12–2.34); q = 0.017	1.92 (0.84–4.37); q = 0.14
Depressive symptoms (PHQ-9 ≥10)	3.19 (1.39–7.35); q = 0.017	2.48 (0.95–6.43); q = 0.094	3.13 (1.35–7.26); q = 0.015	2.55 (1.08–5.99); q = 0.062	3.15 (1.43–6.91); q = 0.014	6.21 (2.50–15.40); q < 0.001
Fatigue (PHQ-9 item 4 ≥2)	2.08 (1.08–4.01); q = 0.043	2.05 (1.06–3.97); q = 0.065	2.32 (1.23–4.37); q = 0.015	2.00 (1.03–3.89); q = 0.062	1.84 (1.16–2.91); q = 0.017	3.46 (1.46–8.21); q = 0.008
Low handgrip strength	1.95 (0.83–4.55); q = 0.147	1.96 (0.83–4.63); q = 0.147	1.99 (0.87–4.55); q = 0.120	2.05 (0.94–4.46); q = 0.085	1.12 (0.64–1.96); q = 0.682	4.92 (1.64–14.78); q = 0.008
Myocardial infarction/angina	0.64 (0.20–2.09); q = 0.460	0.69 (0.21–2.26); q = 0.543	0.62 (0.19–2.03); q = 0.431	0.74 (0.26–2.11); q = 0.572	0.61 (0.32–1.14); q = 0.143	1.29 (0.31–5.34); q = 0.73

Values are odds ratios (95% confidence intervals) with Benjamini–Hochberg false discovery rate-adjusted q values across the six outcomes within each analysis. Columns a–d compare serum magnesium <0.75 vs. ≥0.75 mmol/L.

[a] Models in Table 3 further adjusted for dietary supplement use and antihypertensive medication.

[b] Excluding 31 participants with a urine albumin-to-creatinine ratio (UACR) ≥300 mg/g, to assess the influence of severe albuminuria.

[c] Excluding 4 participants with an estimated glomerular filtration rate (eGFR) <30 mL/min/1.73 m², none of whom had low serum magnesium.

[d] Adjusted for age and sex only.

[e] Serum magnesium <0.85 vs. ≥0.85 mmol/L (≤2.0 mg/dL), with covariates as in Table 3.

[f] Serum magnesium ≤1.7 vs. >1.7 mg/dL (approximately 0.70 mmol/L), with covariates as in Table 3; 44 adults with diabetes had low serum magnesium at this threshold, so estimates for outcomes with few events are imprecise.

PHQ-9, Patient Health Questionnaire-9.

(b) Diagnostic performance of the HbA1c ≥8% or eGFR <60 mL/min/1.73 m² criterion by recorded cut-off used to define low serum magnesium.

Recorded cut-off (mg/dL)	Nominal threshold (mmol/L)	Prevalence in adults with diabetes, %	Diagnostic yield in criterion group, %	Yield ratio vs. all adults with diabetes	Sensitivity, %	Specificity, %	Youden index

≤1.7	0.70	5.0	10.5	2.1	36.7	83.5	0.202
≤1.8 (main analysis)	0.75	10.7	21.7	2.0	35.7	84.6	0.203
≤1.9	0.80	21.8	37.2	1.7	30.1	85.9	0.160
≤2.0	0.85	42.9	55.4	1.3	22.7	86.3	0.089

All values are survey-weighted. Nominal thresholds are the mmol/L values that each recorded cut-off was used to represent; direct conversion of the recorded cut-offs gives 0.70, 0.74, 0.78, and 0.82 mmol/L, and because recorded values are rounded, each classification includes underlying concentrations up to 0.05 mg/dL above the cut-off. Yield ratio is the diagnostic yield in adults with diabetes meeting the criterion divided by the prevalence among all adults with diabetes.

Table S2. Diagnostic performance of guideline-based testing criteria combining HbA1c and eGFR thresholds in adults with diabetes.

**Testing criterion**	***n***	**Adults with diabetes tested, %**	**Diagnostic yield, % (95% CI)**	**Sensitivity, %**	**Specificity, %**	**Youden index**
**HbA1c alone**						
≥7.0%	304	32.9	13.8 (9.9–18.9)	42.5	68.2	0.107
≥7.5%	175	19.4	19.0 (13.0–26.9)	34.5	82.4	0.170
≥8.0%	108	12.4	22.5 (14.5–33.2)	26.2	89.2	0.154
≥9.0%	44	5.7	19.5 (9.3–36.3)	10.3	94.9	0.052
**eGFR alone**						
<90 mL/min/1.73 m^2	448	43.1	12.5 (9.2–16.7)	50.4	57.8	0.081
<60 mL/min/1.73 m^2	76	6.5	21.3 (11.3–36.7)	13.0	94.2	0.073
<45 mL/min/1.73 m^2	21	2.0	39.9 (10.5–79.0)	7.4	98.7	0.061
**HbA1c threshold or eGFR <90 mL/min/1.73 m^2**						
≥7.0%	603	61.6	13.3 (10.4–16.9)	77.0	40.2	0.172
≥7.5%	542	54.6	14.4 (11.1–18.4)	73.5	47.7	0.212
≥8.0%	513	51.2	14.5 (11.1–18.7)	69.4	51.0	0.204
≥9.0%	477	47.0	13.0 (9.7–17.1)	57.1	54.2	0.114

**HbA1c threshold or eGFR <60 mL/min/1.73 m^2**						
≥7.0%	346	36.2	14.8 (11.0–19.6)	50.1	65.4	0.155
≥7.5%	228	23.5	19.6 (14.1–26.5)	43.1	78.9	0.220
≥8.0% (proposed criterion)	171	17.6	21.7 (15.1–30.2)	35.7	84.6	0.203
≥9.0%	115	11.6	18.7 (11.6–28.7)	20.3	89.5	0.098
**HbA1c threshold or eGFR <45 mL/min/1.73 m^2**						
≥7.0%	314	33.6	14.5 (10.6–19.6)	45.8	67.9	0.136
≥7.5%	187	20.2	20.0 (14.1–27.7)	37.8	81.9	0.198
≥8.0%	124	13.7	23.6 (16.0–33.6)	30.4	88.3	0.186
≥9.0%	63	7.2	22.2 (12.8–35.7)	15.0	93.7	0.087

Low serum magnesium was defined as <0.75 mmol/L (recorded value ≤1.8 mg/dL). All values are survey-weighted; *n* is unweighted. The diagnostic yield among all adults with diabetes was 10.7%. Threshold sources: HbA1c 7.0% (general glycemic target, American Diabetes Association), 7.5% (threshold for treatment intensification, NICE), 8.0% (less stringent target, American Diabetes Association), and 9.0% (poor glycemic control, HEDIS); eGFR 90, 60, and 45 mL/min/1.73 m^2 (KDIGO categories G2, G3a, and G3b). Estimates for HbA1c ≥9.0% and eGFR <45 mL/min/1.73 m^2 are based on small numbers and are imprecise. CI, confidence interval; eGFR, estimated glomerular filtration rate; HbA1c, glycated hemoglobin.

Table S3. (a) Prediction models for low serum magnesium (<0.75 mmol/L) in adults with diabetes (*n* = 894; 98 with low serum magnesium).

**Model**	**Variables**	**AUC (95% CI)**	**Optimism-corrected AUC**	**Calibration slope**	**Hosmer–Lemeshow P**
M1	HbA1c, eGFR	0.628 (0.567–0.692)	0.621	0.99	0.89
M2	M1 + age, sex, BMI, insulin use, oral glucose-lowering drug use, antihypertensive drug use	0.668 (0.605–0.726)	0.636	0.81	0.18
M3	M2 + diabetes duration, high-risk drinking, albuminuria	0.705 (0.651–0.763)	0.666	0.80	0.93

(b) Adjusted odds ratios in model M3.

Variable	Odds ratio (95% CI)	P
Age, per year	0.98 (0.95–1.00)	0.055
Female sex	1.33 (0.84–2.12)	0.23
BMI, per kg/m²	1.00 (0.94–1.07)	0.90
HbA1c, per 1%	1.22 (1.01–1.46)	0.037
eGFR, per mL/min/1.73 m²	0.98 (0.97–1.00)	0.011
Insulin use	1.38 (0.57–3.32)	0.47
Oral glucose-lowering drug use	2.14 (1.04–4.43)	0.039
Antihypertensive drug use	0.96 (0.59–1.54)	0.86
Diabetes duration, per year	1.03 (1.00–1.06)	0.053
High-risk drinking	1.82 (0.91–3.64)	0.089
Albuminuria (UACR ≥30 mg/g)	1.84 (1.12–3.04)	0.016

(c) Operating characteristics of model M3 compared with the proposed criterion.

Strategy	Adults with diabetes tested, %	Sensitivity, %	Specificity, %	Positive predictive value, %	Negative predictive value, %
M3, predicted risk ≥11.4% (Youden-optimal)	34.8	63.3	68.7	19.9	93.8
M3, highest-risk 17.2%	17.2	37.8	85.3	24.0	91.8
HbA1c ≥8% or eGFR <60 mL/min/1.73 m²	16.9	33.7	85.2	21.9	91.3

Models were fitted by unweighted logistic regression among adults with diabetes with complete data and internally validated with 500 bootstrap resamples; values in (c) are unweighted. Medication variables are those recorded in KNHANES (insulin use, oral glucose-lowering drug use, and antihypertensive drug use); individual drug classes such as diuretics and proton pump inhibitors are not recorded. High-risk drinking was defined according to the KNHANES definition (≥7 drinks for men or ≥5 drinks for women per occasion, at least twice a week). AUC, area under the receiver operating characteristic curve; BMI, body mass index; CI, confidence interval; eGFR, estimated glomerular filtration rate; HbA1c, glycated hemoglobin; UACR, urine albumin-to-creatinine ratio.
